# Hepatitis C-induced Sjögren’s Syndrome With Positive Serology: A Case Report

**DOI:** 10.7759/cureus.20091

**Published:** 2021-12-01

**Authors:** Adel A Alhazmi, Alhanouf Almuflihi, Mohammed M Aly, Abdelgaffar Mohammed, Abdulrahman Alshehri

**Affiliations:** 1 Department of Internal Medicine, Umm Al-Qura University, Al-Abdia Main Campus, Makkah, SAU; 2 Department of Internal Medicine, Security Forces Hospital, Makkah, SAU; 3 Department of Rheumatology, Security Forces Hospital, Makkah, SAU

**Keywords:** antiviral treatment, extrahepatic manifestation, mixed cryoglobulinemia, sjogren’s syndrome, hepatitis c virus

## Abstract

Sjögren's syndrome (SjS) is a chronic autoimmune disease with a tendency to inflame the exocrine glands, and hepatitis C virus (HCV) infection is considered an exclusion criterion for the diagnosis of this condition; however, it is a highly debated topic, mainly because HCV is viewed as a possible etiopathogenic factor in the disease onset. We report a case of a female patient diagnosed with HCV chronic infection with positive serological markers of SjS (anti-Ro and anti-La autoantibodies). She presented with neuropsychiatric manifestations and casual sicca symptoms and was eventually diagnosed with HCV-induced SjS. Initially, the patient developed symptoms that fulfilled the histopathological criteria of primary SjS and co-existence of mixed cryoglobulinemia, which is viewed as an HCV-related marker.

## Introduction

Sjögren's syndrome (SjS) is a chronic autoimmune disease with a tendency to inflame the exocrine glands, mostly the salivary and lacrimal glands but also the nose, upper respiratory tract, oropharynx, and, in women, the vagina [1]. The primary effect of this inflammation is the development of sicca symptoms, which include mucosal surface dryness, most often in the mouth and eye. Additionally, between one-third and half of the patients may have systemic involvement. SjS has a highly characteristic epidemiological profile during illness manifestation, which may help in the early diagnosis of the condition [2]. SjS disproportionately affects women. In fact, SjS has the most imbalanced gender ratio among all systemic autoimmune disorders; a significant data analysis of more than 14,000 individuals with SjS found a female-to-male ratio close to 10:1 [1]. The absence of effective therapies has been a challenge in the care of patients with primary SjS; nevertheless, awareness about the primary SjS epidemiology has grown, and improvements in establishing categorization criteria, systemic disease activity grading, and patient-reported outcomes have been made in the last decade [2].

We report a case of a female patient diagnosed with hepatitis C virus (HCV) chronic infection with positive serological markers of SjS (anti-Ro and anti-La autoantibodies). The patient presented with neuropsychiatric manifestations and casual sicca symptoms and was later diagnosed with HCV-induced SjS. She initially developed symptoms that fulfilled the histopathological criteria of primary SjS and co-existence of mixed cryoglobulinemia, which is considered an HCV-related marker.

## Case presentation

A 48-year-old woman patient was referred to our hospital's neurology team due to neuropsychiatric manifestation in the form of right-sided old hemiparesis, numbness, left-sided headache, easy fatiguability, and depression. Her condition was associated with dry eye and dry mouth. There were no complaints of fever, fatigue, or any musculoskeletal symptoms. The patient denied any skin manifestation. Other systematic reviews were unremarkable. She had a history of two abortions, one at the 10th week and the other at the 16th week of pregnancy.

The patient underwent a complete blood count (CBC), urine analysis, erythrocyte sedimentation rate (ESR), electrolytes, and liver function test (LFT), and all results were in the normal range. Serological workup showed positive antinuclear antibody (ANA), and anti-SSA/SSB antibodies. Additionally, anti-ds-DNA antibody, anti-smith, complement (C3, C4), direct antiglobulin test (DAT), rheumatoid factor (RF), anti-cyclic citrullinated peptides antibody (anti-CCP), anti-cardiolipin antibody (IgM), anti-cardiolipin antibody (IgG), beta-2 glycoprotein I Ab (IgA), anti-ribonuclear proteins (RNP), anti-scleroderma antigen-70KD (SCL-70), anti-centromere antibody, and anti-histones antibodies were all negative. Also, there were no specific rheumatological symptoms related to Raynaud’s disease, tightness of the skin, or gastroesophageal reflux disease (GERD).

**Figure 1:**
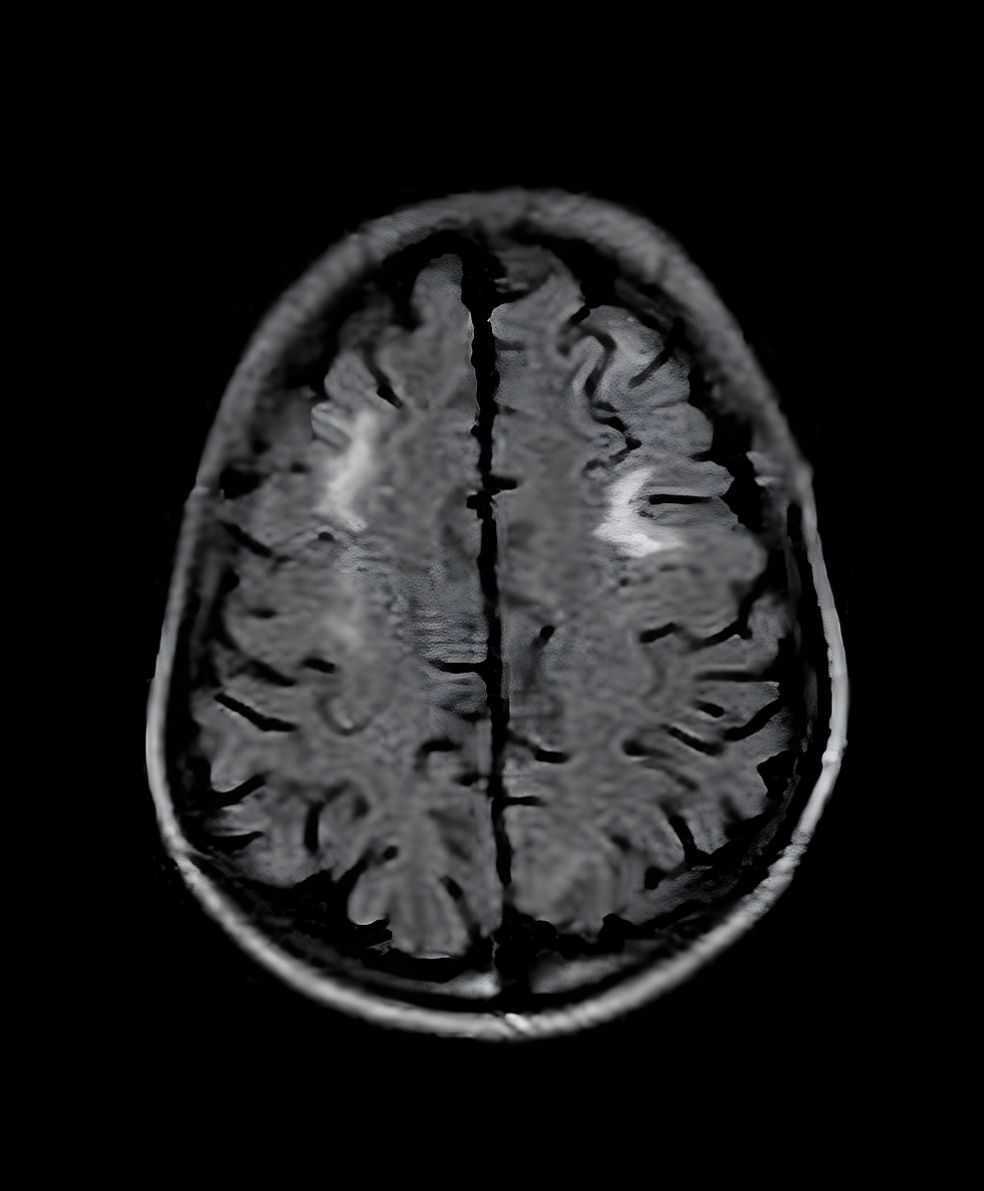
Axial, non-enhanced T1-weighted MRI image demonstrating bilateral multiple juxtacortical white matter high signal intensity lesions MRI: magnetic resonance imaging

A CT was performed to look for the cause of the neurological symptoms, and it was normal. MRI findings (Figure 1) showed bilateral frontal white matter and non-specific bright spots suggesting high signal lesion vasculitis, which correlated with MRA of the cerebral vessels.

Regarding vasculitis, the antineutrophil cytoplasmic antibodies (ANCA) were negative, with the patient symptoms ruling out vasculitis.

According to the American College of Rheumatology (ACR) criteria for the diagnosis of SjS, our patient had eye dryness associated with positive anti-SSA/SSB and an unstimulated whole saliva flow rate of less than 0.1 ml/min with negative labial salivary gland biopsy for lymphocytic infiltration. Based on these findings, she was diagnosed with primary SjS. She was started on steroid and maintenance therapy with azathioprine for four months. 

During her follow-up, the patient had an abnormal LFT. She had elevated levels of alanine aminotransferase (ALT) at 245.4 Unit/L; her aspartate aminotransferase (AST) level was 265u/l, gamma-glutamyl transferase (GGT) was 350.00u/l, and alkaline phosphatase (ALP) was 122.0u/l. 

Accordingly, we ordered serology for HCV, HBV, HIV, and autoimmune hepatitis, which were negative except for HCV; anti-HCV was positive, and HCV RNA-PCR was detected at a rate of 1,964,000. This established the diagnosis of HCV-induced SjS, and hence azathioprine was stopped and the steroid was tapered off until it was eventually discontinued. She was then started on an anti-hepatitis C regimen of ledipasvir-sofosbuvir (LDV/SOF) for three months. The HCV PCR was not detected after three months of therapy, but the positivity for anti-Ro/SS-A, anti-La/SS-B, ANA, and anti-HCV remained. The patient has been currently scheduled for a follow-up.

## Discussion

Our patient had xerophthalmia (eye dryness) associated with positive anti-SSA/SSB and an unstimulated whole saliva flow rate of less than 0.1 ml/min with negative labial salivary gland biopsy for lymphocytic infiltration. Based on these findings, she was diagnosed with primary SjS and treated accordingly. Also, there were no clinical or laboratory indications of HCV at the time of presentation. Nevertheless, the patient was found to be infected with HCV during her clinic follow-up after four months; moreover, she had an abnormal LFT. Hence, three potential conditions were considered in the differential diagnosis: drug-induced hepatitis, autoimmune hepatitis, and viral hepatitis infection. Serological tests revealed reactive anti-HCV, and the diagnosis was confirmed by HCV RNA-PCR detection at a rate of 1,964,000.

HCV infection is a significant cause of chronic liver disease and is considered one of the common causes of mortality worldwide, primarily due to cirrhosis or hepatocellular carcinoma [3]. The global prevalence of HCV has reached 177.5 million people, with the highest number of cases found in Africa, followed by the Middle East. Egypt has the highest prevalence rate of HCV infection (14.7%), and it is 0.7% in Saudi Arabia [4,5].

According to age group distribution, patients between the ages of 20-39 years and 50-69 years had the highest rates of chronic HCV infection, and those aged between 20-39 years were found to have the highest rate of acute HCV infection. In both acute and chronic HCV infections, most of the patients remain asymptomatic, although they can present with fatigue, arthralgia, or jaundice in case of acute infection. Features of decompensated cirrhosis and extra-hepatic manifestations have been reported in chronic HCV infection. Initial diagnosis of HCV is usually established by HCV antibody test accompanied by reflex HCV RNA-PCR [6].

One of the extra-hepatic manifestations of HCV infection is SjS, which presents as the inflammation of the salivary gland. It has been found in 521 out of 38,789 (11.9%) HCV-infected patients [7,8]. The major clinical features and laboratory findings associated with SjS secondary to HCV have been reported as follows: xerostomia, xerophthalmia, positive ocular tests, positive salivary gland biopsy, positive parotid scintigraphy, articular involvement, positive RF, positive ANA, vasculitis, liver involvement, and peripheral neuropathy [9,10]. The ACR has published a new set of criteria for SjS, which concluded that HCV infection is one of the exclusion criteria for primary SjS [11].

Various etiological theories that indicate the association between HCV and SjS have been described in previous studies. Koike et al. have suggested that the HCV envelope protein may exhibit lymphocytes leading to the formation of lymphocytic infiltration in the salivary gland, inducing secondary SjS [12]. Moreover, the HCV RNA was detected in the salivary gland in more than half of the patients (57.5%, n=23/38) in a study by Chernetsova et al. [13]. It is inconsistent with our case; labial salivary gland biopsy was negative for HCV RNA in our patient. Another theory suggests that cryoglobulinemia may play an important role in exhibiting the extra-glandular features of SjS in HCV-infected patients. A higher prevalence of cryoglobulinemia has been found in SjS secondary to HCV when compared to primary SjS [14]. Moreover, one hypothesis has suggested a close association between HLA-DQB1*02 and SjS in chronic HCV patients (p<0.0001) [15]. Unfortunately, no genetic testing was done in the current case.

With regard to cryoglobulinemia, a study suggests that the predominance of cryoglobulinemic-related markers (mixed cryoglobulins, RF, hypocomplementemia) and seronegativity for anti-Ro/SS-A and anti-La/SS-B autoantibodies are essential indicators to differentiate SjS secondary to HCV from primary SjS [16]. However, the present case showed positive anti-Ro/SS-A, anti-La/SS-B, and ANA, and was negative for RF, along with normal levels of complement (C3, C4), and co-existence of mixed cryoglobulinemia.

In previous similar cases, eradication of HCV RNA with anti-hepatitis C medications has resulted in the remission of SjS clinical features [17,18]. Our patient was initially treated as a case of primary SjS, with steroid and azathioprine, according to the European League Against Rheumatism (ELAR) guidelines [19]. She was in remission of SjS symptoms for four months. However, once HCV was detected, azathioprine was stopped and the steroid was tapered off until it was subsequently discontinued. After that, the patient was started on an anti-hepatitis C regimen of LDV/SOF for three months. Three months later, HCV RNA-PCR was not detected, but the positivity for anti-Ro/SS-A, anti-La/SS-B, ANA, and anti-HCV remained.

The current case of HCV-induced SjS exhibited positive serology for anti-Ro/SS-A and anti-La/SS-B. A previous systematic review has shown that the presence of positive SjS serology (anti-Ro/SS-A, anti-La/SS-B) in cases of HCV-induced SjS was found in less than one-fourth of total patients [anti-Ro: 21% (n=29/137), anti-La: 16% (n=22/137)] [9].

## Conclusions

Exclusion of HCV infection is essential before diagnosing primary SjS, even if the patient has no clinical suggestions for HCV infection. HCV infection may induce SjS, and treating HCV infection will be the best course of treatment in such cases. In our patient, the serology for SjS was found to be positive with HCV infection. The current report aimed to raise awareness among clinicians about the need to exclude HCV in SjS cases even when there is positive serology for SjS.
